# Urban professionals’ restorative tourism: exploring the role of perceived environmental restorativeness, push and pull motivations and destination attributes on tourism expectations

**DOI:** 10.3389/fpsyg.2024.1293050

**Published:** 2024-03-19

**Authors:** Xianyao Ding, Jiajun Xu

**Affiliations:** ^1^School of Art and Design, Xihua University, Chengdu, China; ^2^ICT Convergence College of Engineering, Dong-Eui University, Busan, Republic of Korea

**Keywords:** urban professionals, restorative tourism, perceived environmental restorativeness, push and pull motivations, destination attributes, tourism expectations

## Abstract

**Introduction:**

Urban professionals often seek respite from their daily routines through restorative tourism, driven by a complex interplay of motivations that include both internal “push” factors and external “pull” factors. This study investigates the intricate relationship between the perceived environmental restorativeness of tourist destinations and the expectations of urban professionals engaging in restorative tourism. Furthermore, it examines how push and pull motivations mediate this relationship while also considering the moderating effect of destination attributes.

**Methods:**

The multiple regression analyses on the survey data collected from 221 urban professionals with restorative tourism experiences provided quantitative evidence regarding the hypothesized relationships among perceived environmental restorativeness, push and pull motivations, destination attributes, and tourism expectations.

**Results:**

The results showed that perceived environmental restorativeness has a positive effect on urban professionals’ tourism expectations; urban professionals’ pull motivation and push motivation each play a mediating role between perceived environmental restorativeness and tourist expectations; and restorative tourism destination attributes have a moderating effect between perceived environmental restorativeness and push motivation, as well as the relationship between perceived environmental restorativeness and pull motivation.

**Discussion:**

This study provides essential theoretical contributions to restorative tourism and practical implications when designing restorative tourism destinations that target urban professionals.

## Introduction

Stress and anxiety have increased among urban professionals due to exceptional circumstances such as growing job demands, role overload, sedentary lifestyle, unstable employment, and pandemics such as COVID-19 (Lederbogen et al., 2011; Teychenne et al., 2019; Kim et al., 2023). Urban designers and researchers have started to pay attention to preventive measures against the emergence of psychological health issues, such as societal funds for restorative services and infrastructure (Hipp and Ogunseitan, 2011). One type of such service and infrastructure has been greenery and restorative sites in rural and suburban areas (Beil and Hanes, 2013; Van den Berg et al., 2014). Research on restorative environments has demonstrated the advantages of human-nature interactions in various domains (Lehto and Lehto, 2019; Gu et al., 2020; Qiu et al., 2020). In addition, several authors have associated restorative environments with reduced physiological and psychological stress in workplace environments (Pesonen et al., 2011; Lottrup et al., 2013) and hospital settings (Beukeboom et al., 2012). Beautiful natural environments, particularly natural landscapes, are frequently utilized as a resource with psychologically restorative effects (Van den Berg et al., 2007). Connecting with the outdoors, nature, and trees helps individuals escape their daily routines, think about their goals from a different angle, and feel calm and at peace (Lee and Maheswaran, 2011; Vujcic et al., 2019). In particular, after the COVID-19 pandemic, urban professionals have begun to seek a slow and healthy lifestyle (Chi and Han, 2020), with growing tourism demands for weekend getaways and temporary relaxation in rural retreats (Demirovic et al., 2019). Urban professionals’ demand for restorative sites has generated a tremendous market, attracting urban planners and tourism bureaus to provide health and wellness services on greenery sites to attract customers (Xu and Wang, 2023).

Restorative tourism in this study refers to tourism located in a nature-based environment, i.e., a tourism destination that has the ability to promote the recovery of mental resources used by urban professionals to face daily life tasks, thereby contributing to positive outcomes such as cognitive function renewal, stress reduction, an increase in positive emotions, and psychological wellbeing (Hartig, 2011). Indeed, the tourism demands among Chinese urban professionals have recently shifted from mass tourism to restorative tourism (Lehto et al., 2017). Known examples of restorative sites in China include Mountain-to-Sea Trails, which include elevated footpaths and pedestrian bridges to create car-free corridors across the island, allowing urban professionals to escape the hustle and bustle of city life and enjoy a relaxed pace and improving their perceived recovery (Wu et al., 2023). The destinations of restorative tourism are often in domestic locations (Gartner, 2004; Vaishar and Šťastná, 2020; Batista et al., 2022). Many restorative tourism agencies are developing tourism products utilizing local natural and cultural resources to promote heritage tourism, cultural tourism, ecotourism, and vacation tourism (Carrus et al., 2017; Chi and Han, 2021; He et al., 2021). For example, the well-preserved ecological environment, landscape, and folk cultures in the villages serve as the foundation for restorative tourism operations (Nanhua Village, Kaili, Guizhou Province; Likeng Village, Wuyuan, Jiangxi Province) (Gao et al., 2009). Likewise, tourism operations in some historical and cultural towns (Tengchong County and Shun Township) often provide tourist accommodations and various services. Thorough cognitive processing of the environment may occur if the scene piques the interest of enough visitors, which could be accompanied by a decrease in negative emotions (Joye and van den Berg, 2018).

Despite the tremendous market potential, the restorative effect of the cultural environment in the context of the Chinese market has not been evaluated from urban professionals’ perspectives. Existing literature on Chinese restorative tourism consists of qualitative research that focuses on rural tourism’s defining characteristics and promotional value (Wang et al., 2013; Li et al., 2016; Ivona, 2021); yet not much is known about how urban professionals’ perceptions of the promoted characteristics and value of restorative tourism destinations. As such, the purpose of this study is to investigate the relationship between perceived environmental restorativeness and urban professionals’ expectations for tourism, an essential antecedent of tourism behavior (Wong et al., 2013).

Tourism research has sought to understand the driving factors behind why tourists choose to vacation in non-urban areas (Pesonen and Komppula, 2010). According to Šimková and Holzner (2014), Maslow’s five-stage theory, Plog’s psychographic and Iso Ahola’s social psychology are used to analyze tourist motivation. Several studies have investigated the motivations and expectations of rural tourists, but each has focused on tourists in a single destination (Pesonen et al., 2011). For instance, Park and Yoon (2009) highlighted Korea’s diverse restorative tourism industry, addressing visitor needs and expectations and emphasizing relaxation factors such as refreshment, escape, physical activity, and feeling at home. Olsen (2014) found that the motivations and ‘expectation of experience’ of individuals who travel to religious points versus religious lines and religious areas are distinct.

Moreover, different tourism purposes (or route selections) can result in varying tourist expectations that impose different requirements for service design and delivery (Pesonen et al., 2011), which can motivate tourists (Baptista et al., 2020). The ‘push and pull’ factors that affect urban professionals’ perceptions have been considered in a single-factor analysis in a number of studies (Pessoa et al., 2022). These factors include the “forces” that push and pull urban professionals to do so. These forces (motivational factors) explain how motivational variables push people to decide to travel and how the destination area pulls (attracts) them (Baloglu and Uysal, 1996). This study, following Snepenger et al. (2016), divides urban professionals’ motivations into the pull and push factors to contribute to a deeper understanding of the potential impacts of the perceived restorativeness of tourism on tourist expectations. Specifically, this study takes destination attributes into account to identify which destination attributes are more likely to trigger the impact of tourists’ perceived environmental restorativeness on expectations. Thus, this study aims to answer the following questions:

RQ1: What is the mediating mechanism between perceived environmental restorativeness and tourist expectations?

RQ2: How does the attribute of rural tourism destination affect the relationship between perceived environmental restorativeness and rural tourist motivation?

## Literature review

### Restorative environment and stress recovery theory

Restorative time is discretionary time or time spent voluntarily (Staats, 2012). In recent years, there has been a rapid expansion of the literature on restorative environments (Hartig, 2011). Two key theoretical frameworks have emerged in the field of research on the restorative environment: Stress Recovery Theory (SRT) (Ulrich, 1983; Ulrich et al., 1991; McLean et al., 2023) and Attention Restoration Theory (ART) (Kaplan and Talbot, 1983; Kaplan, 1995; Kaplan and Berman, 2010). These two theories explain the restorative effects of a restorative tourism destination. Recent evidence suggests that outdoor, nature-based exposure has a positive effect on various emotional parameters associated with stress relief (Corazon et al., 2019). Exposure to natural environments may restore urban professionals’ ability to concentrate (Stevenson et al., 2018). When exposed to green landscapes, tourists’ directed attention is restored (Jiang et al., 2020). Softly captivating natural landscape elements such as trees, water, and sunsets can draw tourists’ subconscious attention and process quickly, freeing up their directed attention to rest and recoup (Kaplan, 1995). As a result, many tourists choose restorative destinations to reduce their work-related stress. SRT primarily explains how exposure to nature can reduce psychophysiological stress in individuals. As a result, SRT is more relevant to the context of this study. According to SRT (Ulrich et al., 1991), restorative environments can reduce stress (Hunter et al., 2019; Jiang et al., 2020). So far, not many studies have taken culture and the rural environment (attributes) into account. This is despite the fact that destination attributes are critical for the sustainable development of restorative environments. Drawing on the SRT theory, we aim to identify the motivations and consequences of tourists who feel work-related stress and often travel to restorative environments.

### Motivation: pull and push

Push and pull factors are a commonly used framework for studying tourist motivations. Based on the idea that people travel because they are internally and externally motivated by the characteristics of their destination, this framework of two-dimensional forces acts as motivating elements for tourists (Otoo and Kim, 2018). In general, the pull factors of a rural tourism destination cause urban professionals to feel drawn there. Natural attractions, cultural resources, recreational opportunities, special events, and festivals are frequently used as pull factors (Kim and Lee, 2002). Additionally, push factors include the needs of urban professionals for their travel (Caber and Albayrak, 2016). Push factors may include urban professionals’ mental health, which may influence their desire to engage in particular tourism activities. In addition, a sense of necessity or an urge can drive tourists to perform specific actions (Xue et al., 2022). Pull and push forces ultimately enable urban professionals to make well-informed decisions regarding their travel arrangements and ensure a fulfilling and rewarding experience (Prayag and Ryan, 2011). In this study, restorative tourism destinations represent a unique resource and objective in a particular market to satisfy a variety of tourist motivations and exceed their expectations.

### Perceived environmental restorativeness and tourist expectations

Research on urban stressors (Glass and Singer, 1972) has suggested that living in cities can create urban professionals’ restoration needs. Safety, health, and the pursuit of happiness are crucial factors for rural tourism organizers when promoting tourist destinations (Robina-Ramirez et al., 2023), especially during the COVID-19 pandemic, when personalized restorative tourism grew significantly compared to mass tourism (Batista et al., 2022). With the end of the pandemic, urban professionals’s retaliatory consumption patterns have contributed to the recovery of restorative tourism. The pressure accumulated daily, especially employment and work pressure during the pandemic, can be alleviated through rural tourism, increasing tourists’ expectations (Abdou et al., 2022). Prior studies have distinguished between the activity itself and work-related stress relief (Manfredo et al., 2017; Witt and Bishop, 2017). In short, the following hypothesis can be developed:

*H1*: Perceived environmental restorativeness has a positive effect on tourist expectations.

### The mediating effects of pull and push motivations

According to Saxena and Ilbery (2008), restorative tourism is primarily supported by social networks that explicitly link local stakeholders to promote and maintain the economic, social, cultural, natural, and human resources of the destination (Wang et al., 2013). Several restorative tourism segmentation studies have investigated what motivates urban professionals to visit rural areas. Frequently, push and pull motivations are included in the same factor analysis, especially in benefit segmentation studies (Pesonen et al., 2011). Bansal and Eiselt (2004) argued that tourist motivations and the image of all regions and travel companions influence region selection. Urban professionals decide on their vacation destination based on what the tourism destination has to offer, i.e., pull motivations, after they have made detailed plans for their trip. As tourists’ life stage develops, push motives may change in significance, becoming relatively homogeneous due to declining health and immobility (Lewis and D'Alessandro, 2019). Moreover, urban professionals choose rural tourism because it will help them manage the stress of working in cities (push factors).

Additionally, many young urban professionals are attracted by the idyllic pastoral lifestyle. Tourist expectations in numerous tourism fields, including rural tourism, include relaxation, natural landscape, fresh air, and rural tranquility (Perales, 2002; Devesa et al., 2010; Chi and Han, 2021). Previous studies on the mediating role of motivation have focused on the attributes of tourist destinations as pull factors, using mostly keywords with destination characteristics as data collection codes (Crompton, 1979; Dann, 1981). However, there needs to be a unified theoretical interpretation for universal understanding. This study collected data from different tourist destinations in China using a questionnaire survey, attempting to explain the entire phenomenon using theory. Thus, the following hypotheses are as follows:

*H2*: Pull motivation plays a mediating role between perceived environmental restorativeness and tourist expectations.

*H3*: Push motivation plays a mediating role between perceived environmental restorativeness and tourist expectations.

### The moderating effect of the restorative tourism destination’s attributes

Prior research suggested comparing destination attributes between segments to provide rural tourism organizers with more options for differentiating their offerings and increase academic understanding of the interaction between push and pull motivations (Pesonen, 2012; Amoah et al., 2018; Trimurti and Utama, 2021). Few studies have performed a comprehensive empirical analysis of destination attributes, thus limiting our understanding of the moderating effects of a restoraitve tourism site’s attributes. The tourism literature has documented two essential destination attributes, i.e., pro cultural heritage and pro natural. While pro cultural heritage tourism destinations attract tourists with the tangible and intangible heritage of an area through experiences or activities that associate specific individuals, objectives, and places with these destinations (Hernández-Rojas et al., 2021; Dabphet, 2023), pro natural tourism destinations attract tourists with responsible travel experiences in natural areas with specific landscape, flora and fauna while protecting the environment and improving the quality of life of locals (Huybers and Bennett, 2003; Sørensen and Grindsted, 2021). These two attributes can attract tourists differently. These two attributes can help understand how the perceived restorative aspects of these destinations influence tourists’ motivations, thereby examining whether and how tailored tourism experiences and cultural and natural resource reservations can contribute to tourism destination development. Therefore, this study adopts these two destination attributes as moderating variables to determine which types of landscapes influence which motivations (pull & push factors). Hence, the following hypotheses are formulated:

*H4*: Restorative tourism destination attributes have a moderating effect between perceived environmental restorativeness and pull motivation. Thus, pro cultural heritage destination has more effect on pull motivation than pro natural destination.

*H5*: Restorative tourism destination attributes have a moderating effect between perceived environmental restorativeness and push motivation. Thus, pro cultural heritage destination has more effect on push motivation than pro natural destination.

**Figure 1:**
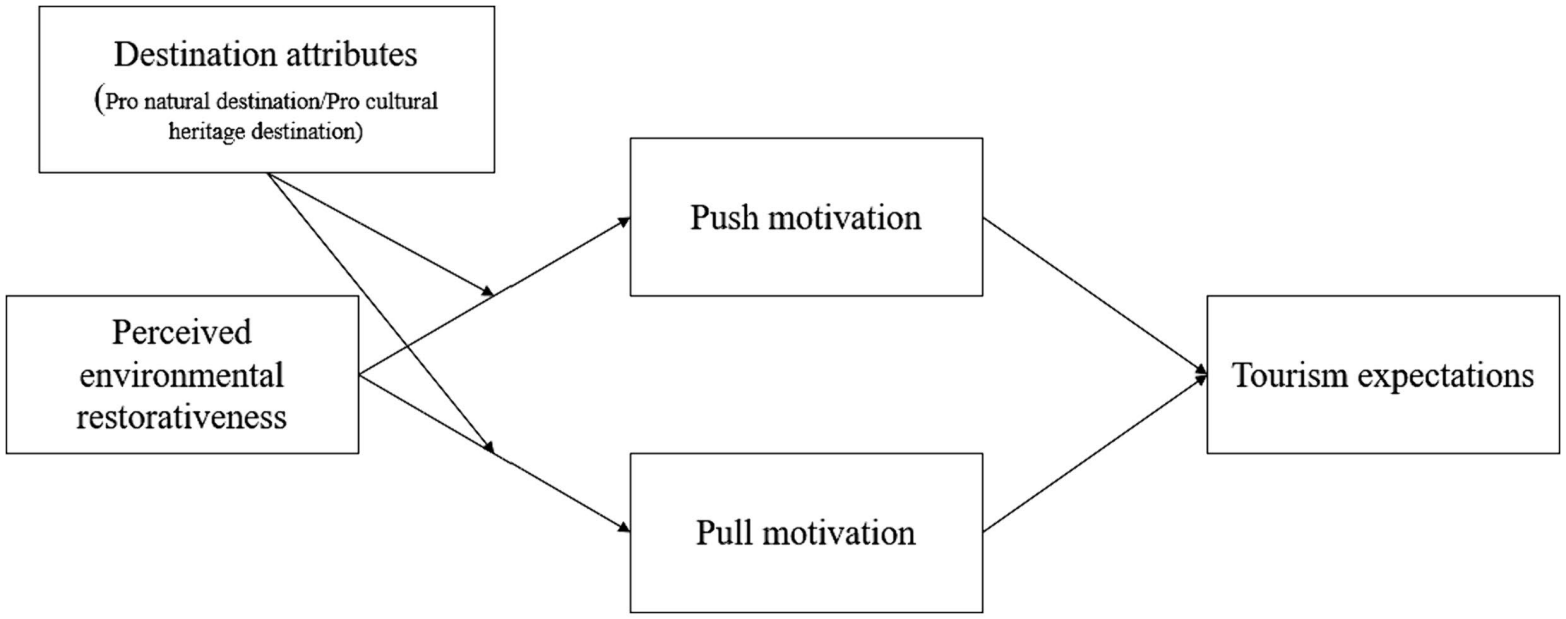
Conceptual model.

The conceptual model is presented as follows Figure 1.

## Methods

### Sampling

This study examined the hypothesized relationships by surveying rural tourists from China. The survey employed the online survey platform ‘WENJUANXING’ (a questionnaire survey platform widely used in China). This study collected 253 responses; after excluding 32 invalid questionnaires, the sample for analysis consisted of 221 responses (87.35% of the total). 53.4% of the respondents are male (*n* = 118), and 46.6% of the respondents are female (*n* = 103). 26–30 years old (33.9%) has the highest per cent among the five groups, followed by 31–35 years old (33.0%), 41 years old and above (11.8%), 20–25 years old (11.3%) and 36–40 years old (10.0%). In attributes of destinations, pro natural destination accounts for 45.2% (*n* = 100), pro cultural heritage destination accounts for 54.8% (*n* = 121). Table 1 presents the results of those respondents.

**Table 1:** Demographic description.

Variable		Frequency	Percentage
Gender	Male	118	53.4%
	Female	103	46.6%
Age	20–25 years old	25	11.3%
	26–30 years old	75	33.9%
	31–35 years old	73	33.0%
	36–40 years old	22	10.0%
	41 years old and above	26	11.8%
Destination attributes	Pro natural destination	100	45.2%
	Pro cultural heritage destination	121	54.8%

### Measures

This study included four constructs: perceived environmental restorativeness (mentally away, physically away), pull motivation, push motivation, and tourist expectations. This study translated measurement items into Chinese to better utilize our Chinese respondents. Back-translation ensured that each item’s meaning and context were consistent. The items were translated into Chinese by one author and back into English by another. This approach allowed us to maintain semantic equivalence throughout the translation process. A 5-point Likert scale was used for each measurement item.

Minor modifications were made to all previously used measurement items to account for the context of perceived environmental restorativeness in Chinese tourism destinations. Perceived environmental restorativeness was adapted from Chen et al. (2017): mentally away (4 items) and physically away (3 items). Pull motivation and push motivation were adapted from Robina-Ramirez et al. (2023) (pull motivation: 3 items, push motivation: 3 items). Tourist expectations were adapted from Narangajavana et al. (2017) (3 items).

### Research method

This study adopted the SPSS and AMOS packages to execute data analysis. Simultaneously, the constructs of the research model were evaluated for their reliability and validity. Confirmatory factor analysis (CFA) was used to test the measurement model for the goodness of model fit, discriminant and convergent validity. Direct, indirect and moderating effects were tested in the PROCESS macro in SPSS to examine the significance of the path coefficients and confirm the research hypothesis.

## Research results

### Assessment of the measurement model

This study first conducted a confirmatory factor analysis in AMOS. The reliability and validity were first examined to test the measurement model. As shown in Table 2, the factor loadings of the items are all higher than the required 0.70. Cronbach’s alpha and composite reliability (CR) were used to evaluate internal consistency reliability. The Cronbach’s Alpha (from 0.794 to 0.900) and the CR (from 0.796 to 0.901) of each construct are larger than the required 0.70 level, confirming the internal consistency and reliability. The model fit of the measurement model showed that χ2/df (chi-square divided by the value of the degree of freedom) = 1.149, CFI (comparative fit index) = 0.993, TLI (Tucker–Lewis index) = 0.991, RMR (root-mean-square residual) = 0.044, RMSEA (root mean square error of approximation) = 0.026, indicating a good fitness of the collected data with the measurement model. Average variance extracted (AVE) was utilized to evaluate convergent and discriminant validity. As shown in Table 2, all of the AVE values are greater than the required 0.5 level (0.566 to 0.725), confirming the convergent validity (Fornell and Larcker, 1981). According to Table 3, the correlations between the two constructs are all less than the square root of the construct’s AVE, which satisfies the criterion and confirms the discriminant validity.

**Table 2:** Results of reliability and validity.

Constructs	Item	Factor loading	CR	AVE	Cronbach’s Alpha
Mentally away	MA1	0.804	0.901	0.694	0.900
	MA2	0.887			
	MA3	0.844			
	MA4	0.794			
Physically away	PA1	0.824	0.796	0.566	0.794
	PA2	0.701			
	PA3	0.727			
Pull motivation	PL1	0.837	0.847	0.649	0.846
	PL2	0.833			
	PL3	0.744			
Push motivation	PH1	0.803	0.888	0.725	0.885
	PH2	0.822			
	PH3	0.925			
Tourist expectations	TE1	0.821	0.883	0.716	0.879
	TE2	0.867			
	TE3	0.850			

**Table 3:** Results of correlations and discriminant validity.

Constructs	Mean	SD	1	2	3	4	5	6	7	8
1. Gender	1.466	0.500	--							
2. Age	2.769	1.147	0.077	--						
3. Attribute	1.548	0.499	0.066	0.174^**^	--					
4. MA	3.457	1.079	0.016	0.160^*^	0.183^**^	0.833				
5. PA	3.733	1.030	−0.054	0.076	0.215^**^	0.417^**^	0.753			
6. PL	3.893	0.848	0.068	0.157^*^	0.225^**^	0.368^**^	0.271^**^	0.806		
7. PH	3.701	1.016	0.013	0.125	0.390^**^	0.391^**^	0.314^**^	0.393^**^	0.852	
8. TE	3.549	1.032	0.074	0.264^**^	0.214^**^	0.407^**^	0.323^**^	0.430^**^	0.435^**^	0.846

*, *p* < 0.05; **, *p* < 0.01; MA, mentally away; PA, physically away; PL, pull motivation; PH, push motivation; TE, tourist expectations.

### Hypothesis testing

The data were examined for direct and indirect effects using SPSS macro model 4. As shown in Table 4, PRQ has a significant positive effect on TE (*B* = 0.271; LLCI = 0.125, ULCI = 0.417); PL has a mediating effect on the relationship between PRQ and TE (*B* = 0.089; LLCI = 0.024, ULCI = 0.171); PH has a mediating effect on the relationship between PRQ and TE (*B* = 0.094; LLCI = 0.027, ULCI = 0.164). Thus, H1, H2, and H3 are supported.

**Table 4:** Results of direct and indirect effects.

Path	Effect	se	LLCI	ULCI
PRQ-TE	0.271	0.074	0.125	0.417
PRQ-PL-TE	0.089	0.038	0.024	0.171
PRQ-PH-TE	0.094	0.035	0.027	0.164

PRQ, perceived environmental restorativeness; PL, pull motivation; PH, push motivation; TE, tourist expectations; LLCI, lower limit of 95% confidence interval; ULCI, upper limit of 95% confidence interval.

SPSS macro Model 1 was utilized for moderating effects testing. According to Table 5, the interaction of PRQ and Attributes has a significant positive effect on pull motivation (*B* = 0.269; LLCI = 0.028, ULCI = 0.509). The specific moderating effect can be determined from Table 6. As demonstrated in Table 6, pro cultural heritage destination has more effect (*B* = 0.476; LLCI = 0.296, ULCI = 0.656) on pull motivation than pro natural destination (*B* = 0.207; LLCI = 0.047, ULCI = 0.367). Thus, H4 is supported.

**Table 5:** Results of the moderating effect on pull motivation.

DV: pull motivation	B	se	*t*	*p*	LLCI	ULCI
constant	3.195	0.243	13.134	0.000	2.716	3.675
PRQ	−0.062	0.186	−0.332	0.741	−0.428	0.305
Attributes	0.214	0.109	1.976	0.050	0.001	0.428
PRQ*Attributes	0.269	0.122	2.204	0.029	0.028	0.509
Gender	0.128	0.105	1.216	0.226	−0.079	0.335
Age	0.055	0.047	1.173	0.242	−0.037	0.146

PRQ, perceived environmental restorativeness; Attributes, destination attributes; LLCI, lower limit of 95% confidence interval; ULCI, upper limit of 95% confidence interval.

**Table 6:** Conditional effects of perceived environmental restorativeness on pull motivation.

DV: pull motivation	B	se	*t*	*p*	LLCI	ULCI
Pro natural destination	0.207	0.081	2.554	0.011	0.047	0.367
Pro cultural heritage destination	0.476	0.091	5.219	0.000	0.296	0.656

LLCI, lower limit of 95% confidence interval; ULCI, upper limit of 95% confidence interval.

According to Table 7, the interaction of PRQ and Attributes has a significant positive effect on push motivation (*B* = 0.349; LLCI = 0.078, ULCI = 0.620). The specific moderating effect can be determined from Table 8. As demonstrated in Table 8, pro cultural heritage destination has more effect (*B* = 0.589; LLCI = 0.387, ULCI = 0.792) on push motivation than pro natural destination (*B* = 0.241; LLCI = 0.060, ULCI = 0.421). Thus, H5 is supported.

**Table 7:** Results of the moderating effect on push motivation.

DV: push motivation	B	se	*t*	*p*	LLCI	ULCI
Constant	2.629	0.274	9.595	0.000	2.089	3.169
PRQ	−0.108	0.210	−0.516	0.606	−0.521	0.305
Attributes	0.620	0.122	5.068	0.000	0.379	0.860
PRQ*Attributes	0.349	0.137	2.538	0.012	0.078	0.620
Gender	0.028	0.118	0.239	0.812	−0.205	0.261
Age	0.013	0.052	0.251	0.802	−0.090	0.116

PRQ, perceived environmental restorativeness; Attributes, destination attributes; LLCI, lower limit of 95% confidence interval; ULCI, upper limit of 95% confidence interval.

**Table 8:** Conditional effects of perceived environmental restorativeness on push motivation.

DV: push motivation	B	se	*t*	*p*	LLCI	ULCI
Pro natural destination	0.241	0.091	2.633	0.009	0.060	0.421
Pro cultural heritage destination	0.589	0.103	5.736	0.000	0.387	0.792

PRQ, perceived environmental restorativeness; Attributes, destination attributes; LLCI, lower limit of 95% confidence interval; ULCI, upper limit of 95% confidence interval.

## Discussion

This research uniquely contributes to the growing body of research in restorative tourism in rural areas (Li et al., 2016; Ivona, 2021) from a unique cohort of consumers, i.e., urban professionals. Such a perspective is important because urban professionals living in stressful work conditions (Kim et al., 2023) form a lucrative market for restorative tourism in rural areas. In this unique context, this study constructed and tested a conceptual model of urban professionals’ expectations based on the stress recovery theory (Ulrich et al., 1991). Our results on the impact of perceived environmental restorativeness on urban professionals’ travel expectations through push-pull motivation (internal needs of tourists and external needs of the destination) concur with previous studies (Hunter et al., 2019; McLean et al., 2023) on the explaining power of this theory. In particular, we found that urban professionals’ perceived environmental restorativeness is positively associated with their tourism expectations. Based on the findings, urban professionals are eagerly anticipating the restorative effects of restorative tourism; in particular, the landscapes, natural environment, architecture, and culture of rural areas seem to provide the push and pull motivations that form the ‘expectation of experience’ discussed in the literature (Olsen, 2014).

We also find that urban professionals’ push and pull motivations significantly mediate the relationship between perceived environmental restorativeness and tourist expectations. This indicates that perceived environmental restorativeness is crucial for the internal and external needs of the destination in rural tourism. Tourists are motivated to travel due to their internal needs as well as the promotion of rural tourism destinations. Such a mediating mechanism extends previous studies (Bansal and Eiselt, 2004; Chi and Han, 2021; McLean et al., 2023) that either focus on the internal (e.g., life stage) or the external (e.g., destination image and characteristics) motivations of tourists.

Moreover, we proved the moderating effects of destination attributes on the relationship between perceived environmental restorativeness, push motivation, and pull motivation. Our results concur with previous studies on the significance of differentiating between two destination attributes: pro cultural heritage destinations and pro natural destinations (Sørensen and Grindsted, 2021; Dabphet, 2023). More importantly, this study adds these studies by elaborating on how each destination attribute contributes differently to urban professionals’ motivations toward restorative tourism.

### Theoretical contributions

The findings of this study make several theoretical contributions. First, our study explains why perceived environmental restorativeness affects tourists’ expectations from the perspective of push-pull motivation. This distinction answers the call (Olsen, 2014) to examine tourists’ different motivations when traveling to a specific destination, and extends previous studies (Olsen, 2014) that consider the push-pull motivation as a single analysis. We empirically verified push-pull motivation as the mechanism mediating the relationship between perceived environmental restorativeness and urban professionals’ tourism expectations. In doing so, we explained how destination-generated factors, such as nature and greenery, and urban professionals’ internal factors, such as life stage, collectively shape urban professionals’ expectations for restorative tourism destinations.

In the unique context of urban professionals’ expectations of tourist experience in non-urban areas, this study examines how restorative tourism destinations are considered to be beneficial by urban professionals who are internally attracted by stress reduction. In doing so, this finding provides empirical evidence on previous studies regarding the market demand for restorative tourism (Hipp and Ogunseitan, 2011; Qiu et al., 2020). Therefore, our research provides a theoretical basis for future research on rural restorative environments.

Second, our research focuses on perceived environmental restorativeness, a novel approach in restorative tourism research (Park and Yoon, 2009) that primarily focuses on relaxation factors. While environmental restorativeness is an effective form of relaxation for urban professionals (Li et al., 2016), it is necessary to consider how those professionals perceive the various elements provided by restoriative tourism destinations to be truly motivationing, and thus form the expectations. Therefore, our research has raised the awareness of integrating potential consumers’ preferences when designing attributes of rural tourism destinations (Chi and Han, 2021). Thus, our study contributes to the existing literature on rural tourism.

Third, this study examined the moderating effect of destination attributes. In previous studies (Kim and Lee, 2002; Otoo and Kim, 2018), destination attributes were either tested as a type of pull motivation or classified as urban and rural (Amoah et al., 2018). In this study, based on the unique context of urban professionals’ perception of restorative tourism in China, destination attributes are classified as pro-natural and pro-cultural heritage. We extend previous studies (Trimurti and Utama, 2021) that link destination attributes to tourist behavior and expectations by unraveling the mediating path of tourist motivation. In particular, we find that destination attributes closer to the natural environment with cultural heritage are more likely to promote the generation of tourist motivation than destination attributes more relative to the natural environment. This finding provides a new perspective on the impact of destination attributes.

### Practical contributions

The findings of this study also provide restorative tourism planners and local tourism bureaus with practical suggestions.

First, our results show that the perceived environmental restorativeness can positively influence urban professionals’ expectations through push-pull motivation. Therefore, restorative tourism planners and local tourism bureaus who have not promoted regional tourism characteristics should attract the targeted customers (e.g., urban professionals) by establishing the pulling elements related to their restorative needs. For instance, planners can adopt a variety of advertising channels to demonstrate the various restorative services and benefits to urban professionals and to communicate with and guide them, thereby fostering perceived environmental restorativeness. Restorative tourism planners and local tourism bureaus who have already promoted regional tourism characteristics should investigate and understand the unhealthy factors, such as accumulated stress in urban professionals’ work and life, and design experiences and services that can alleviate those feelings. In addition, local tourism bureaus can enhance government support policies for restorative tourism areas and increase investment in local infrastructure, thereby enhancing their expectations.

Second, restorative tourism planners should promote the destination’s cultural heritage in order to differentiate themselves from other tourism destinations. For instance, if urban professionals have a greater internal demand for cultural heritage, then planners should focus on promoting the local natural environment with pro-cultural heritage, such as good air quality and ancient relics, so that urban planners can not only relax physically but also appreciate the local history and culture.

### Limitations and future research

Several limitations exist in our study. First, there is a paucity of empirical literature on the perceived environmental restorativeness of the environment, and this study does not discuss its antecedent variables. We propose that empirical research on the antecedent variables of perceived environmental restorativeness can be conducted in the future. Second, regarding the categorization of destination attributes, this study only distinguished between two groups: pro-natural environment destination attributes and pro-cultural heritage destination attributes. Due to the increase in various types of rural tourism destinations in China in recent years, we recommend that future research refine the classification of destination attributes and cover them as thoroughly as possible.

## Data availability statement

The raw data supporting the conclusions of this article will be made available by the authors, without undue reservation.

## Author contributions

XD: Writing – original draft, Writing – review & editing. JX: Writing – original draft, Writing – review & editing.
